# Trends in lung cancer incidence by age, sex and histology from 2012 to 2025 in Catalonia (Spain)

**DOI:** 10.1038/s41598-021-02582-8

**Published:** 2021-12-02

**Authors:** Laura Guarga, Alberto Ameijide, Rafael Marcos-Gragera, Marià Carulla, Joaquim Delgadillo, Josep Maria Borràs, Jaume Galceran

**Affiliations:** 1 Servei Català de la Salut (CatSalut), Barcelona, Spain; 2 Departament de Farmacologia, Universitat Autònoma de Barcelona, Cerdanyola del Vallès, Barcelona, Spain; 3 Registre de Càncer de Tarragona, Servei d’Epidemiologia i Prevenció del Càncer, Hospital Universitari Sant Joan de Reus, IISPV, Reus, Tarragona Spain; 4 Pla Director d’Oncologia, Departament de Salut, Barcelona, Spain; 5 Unitat d’Epidemiologia i Registre del Càncer de Girona (UERGG), Institut d’Investigació Biomèdica Girona Josep Trueta (IDIBGI), Girona, Spain; 6 Biomedical Network Research Centers of Epidemiology and Public Health (CIBERESP), Madrid, Spain; 7 Departament d’Infermeria, Universitat de Girona (UdG), Girona, Spain; 8 Banc de Sang i Teixits (BST), Barcelona, Spain; 9 Departament de Ciències Clíniques, Universitat de Barcelona, Campus de Bellvitge, L’Hospitalet de Llobregat, Barcelona, Spain; 10 Departament de Medicina i Cirurgia, Universitat Rovira i Virgili, Reus, Tarragona Spain

**Keywords:** Cancer, Epidemiology

## Abstract

Lung cancer remains one the most common cancers in Europe and ranks first in terms of cancer mortality in both sexes. Incidence rates vary by region and depend above all on the prevalence of tobacco consumption. In this study we describe recent trends in lung cancer incidence by sex, age and histological type in Catalonia and project changes according to histology by 2025. Bayesian age-period-cohort models were used to predict trends in lung cancer incidence according to histological type from 2012 to 2025, using data from the population-based Catalan cancer registries. Data suggest a decrease in the absolute number of new cases in men under the age of 70 years and an increase in women aged 60 years or older. Adenocarcinoma was the most common type in both sexes, while squamous cell carcinoma and small cell carcinoma were decreasing significantly among men. In both sexes, the incident cases increased by 16% for patients over 70 years. Increases in adenocarcinoma and rising incidence in elderly patients suggest the need to prioritize strategies based on multidisciplinary teams, which should include geriatric specialists.

## Introduction

Lung cancer remains one of the most common incident cancers in Europe, ranking first in terms of cancer mortality in both sexes. In 2020, the estimated number of new cases in Spain was 29,188 and the number of estimated deaths 22,930, with the European age-standardized mortality rate of 47.6 per 100,000 habitants^1^. Its impact in Spain has differed greatly between men, in whom incidence is decreasing, and in women, in whom it has been rising quite sharply from a very low incidence rate^2,3^. Lung cancer has low survival rates, hovering around 15% in high-income countries worldwide^4^ though some differences are observed across histologic type^5^.

The evolution in lung cancer incidence has also been accompanied by changes in the relevance of different histological types. In one comparative analysis, trends in adenocarcinoma were shown to be increasing in women but stabilizing in men across many high-income countries^6^. The overall decrease in lung cancer incidence was mainly driven by reductions in squamous cell carcinoma in men^6^ and by the decline in the prevalence of smoking^7^. However, in that analysis, Spain showed a distinct pattern, with the squamous type still dominant among men, similar to the pattern in France. These divergent trends could have therapeutic implications due to the differential treatment approaches by histological type and location^8^.

Indeed, targeted systemic therapies for lung cancer are increasingly being developed for different histologies, which are usually characterized by specific biomarkers^9,10^. These may have a predictive effect on which treatment strategy is followed^9,10^. Currently known biomarkers correspond to mutations in *EGFR*, *BRAF METex14* and *KRAS*; rearrangements or fusions affecting *ALK*, *ROS1* and *RET*; and the ligand expression of programmed cell death protein (PD-L1)^9,10^. Data on biomarker prevalence at the population level are not available, posing challenges for planning the provision of the most appropriate therapeutic approaches in coming years.

A better understanding of the trends in lung cancer incidence could help predict changes in the age and histological profile in a given population, which is relevant for health services organization and treatment resource planning^11,12^. In light of the changes in histology by sex and the resulting implications for evaluating treatment needs, this study aims to assess incidence trends in lung cancer according to age, sex, and histology, and to make projections on incidence in Catalonia to 2025.

## Methods

Cancer incidence data for the period 1994–2012 were provided by the Girona^13^ and Tarragona^14^ population-based cancer registries. Population data for the provinces of Girona and Tarragona and for the Catalonia as a whole were provided by the Catalan Institute of Statistics^15^.

According to the International Classification of Diseases for Oncology, 3rd edition (ICD-O-3), lung cancer cases were classified into the following histological types: non-small cell carcinoma, including squamous cell carcinoma (8052, 8070–8076, 8083–8084, 8123), adenocarcinoma (8140, 8200, 8211, 8260, 8230, 8255, 8310, 8323, 8430, 8480, 8481, 8490, 8550, 8560, 8570), and large and other non-small cell cancers (8012, 8046, 8250–8253); small cell carcinoma (8041–8045); and unspecified/carcinoma not otherwise specified and others (8000, 8001, 8010, 8031, 8020–8022, 8033, 8082, 8800, 8815, 8980, 9140, 9220, 9530). Neuroendocrine tumors were not included in the study.

Lung cancer (International Classification of Diseases, 10th revision-ICD-10-C34) projections for the years 2020 and 2025 were obtained for Catalonia by five-year age group using a Bayesian autoregressive age-period-cohort (APC) model that assumes a Poisson distribution for new diagnoses^2^. The projection of rates and new cases for Catalonia from 2019 to 2025 were obtained by five-year age group by interpolating the projected rates for the years 2020 and 2025.

The estimated lung cancer incidence in Catalonia for 2012 was obtained by applying the pooled rates of Tarragona and Girona for the period 2010–2014. The lung cancer mortality ratio was calculated as the lung cancer mortality rate in Catalonia, on the one hand, and in Tarragona and Girona on the other, both based on data from 2010–2014 and adjusted according to the new European population pyramid. The projection of rates and new cases for Catalonia from 2013 to 2019 were obtained by five-year age group by interpolating the projected rates for the years 2012 and 2020.

Based on data of lung cancer incidence by histological type in Tarragona and Girona for the 2005–2014 period, we used a logistic regression model to estimate the percentage that each of the histological types represents in relation to total cases of lung cancer according to sex, year of diagnosis and age group, and we applied these percentages to the total number of estimated cases to estimate the number of cases and rates of lung cancer by histological type in 2012–2025.

**Figure 1:**
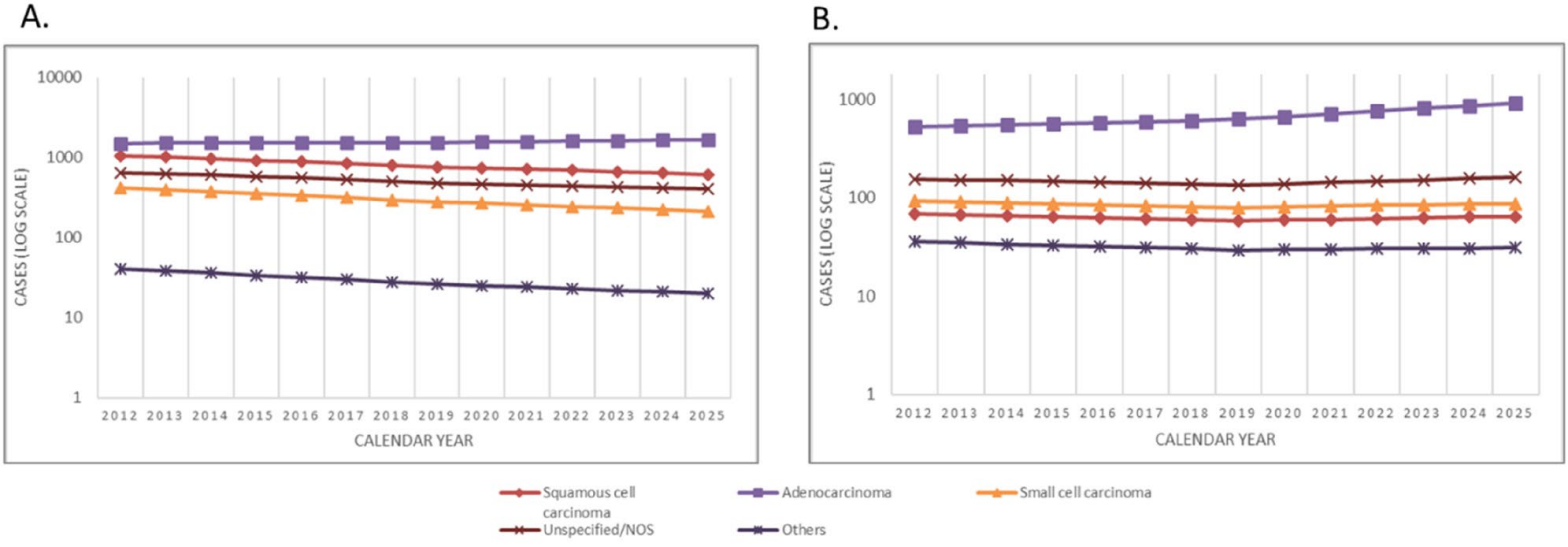
Incidence of lung cancer in men (**A**) and women (**B**) in Catalonia between 2012 and 2025 by histology. NOS: not otherwise specified. Others: This group contains several histological types of low frequency. Note: These figures reflect trends in the number of new lung cancer cases per year.

**Figure 2:**
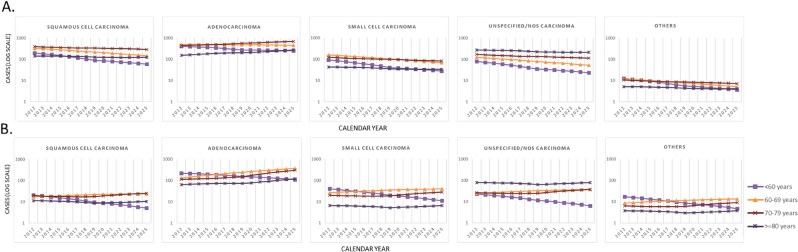
Incidence of lung cancer in men (**A**) and women (**B**) in Catalonia between 2012 and 2025 by histology and age group. NOS: not otherwise specified. Others: This group contains several histological types of low frequency. Note: These figures reflect trends in the number of new lung cancer cases per year by histology and sex. The age group (< 60 years) includes 0 to 60 years.

For the years 2012 and 2025, the number of new cancer cases, the difference in cases between 2020 and 2015, the crude rate (CR) and the age-standardized incidence rate to the new European population (ASIR_E_) by sex and age group (< 60; 60–69; 70–79 and ≥ 80) (Table 1) and by histological type (Table 2) are shown, as is the evolution of ASIR by histological type (Fig. 1) and stratified by sex and age group (Fig. 2).

**Table 1:** Differences in the number of incident lung cancer in Catalonia between 2012 and 2025 by age group.

Age group	2012		2025		Differences^a^	Net changes^b^	CR 2012	CR 2025	ASIR 2012	ASIR 2025
	N	[95% CI]	N	[95% CI]	N	%				
**Men**										
< 60 years	797	[742; 852]	369	[331; 407]	− 428	− 53.7%	26.97	12.76	29.88	11.50
60–69 years	1103	[1038; 1168]	726	[673; 779]	− 377	− 34.2%	305.53	162.12	306.56	163.89
70–79 years	1150	[1084; 1216]	1177	[1110; 1244]	27	2.3%	481.75	380.05	479.73	378.57
≥ 80 years	615	[566; 664]	656	[606; 706]	41	6.6%	430.56	347.35	414.36	345.03
Total	3666	[3547; 3785]	2928	[2822; 3034]	− 738	− 20.1%	99.13	76.27	121.41	78.74
**Women**										
< 60 years	323	[288; 358]	139	[116; 162]	− 184	− 57.0%	11.35	4.70	11.75	4.31
60–69 years	206	[178; 234]	489	[446; 532]	283	136.9%	52.28	99.90	52.27	100.03
70–79 years	187	[160; 214]	404	[365; 443]	217	115.7%	63.19	107.01	62.41	107.43
≥ 80 years	166	[141; 191]	221	[192; 250]	55	33.3%	63.52	71.36	63.44	71.31
Total lung cancer	883	[825; 941]	1253	[1184; 1322]	370	42.0%	23.24	31.24	23.55	27.95
**Both sexes**										
< 60 years	1121	[1055; 1187]	508	[464; 552]	− 613	− 54.7%	19.31	8.87	20.82	7.90
60–69 years	1310	[1239; 1381]	1215	[1147; 1283]	− 95	− 7.2%	173.27	129.63	179.41	131.96
70–79 years	1338	[1266; 1410]	1581	[1503; 1659]	243	18.2%	249.93	230.05	271.07	243.00
≥ 80 years	781	[726; 836]	877	[819; 935]	96	12.3%	193.42	175.91	238.90	208.17
Total lung cancer	4549	[4417; 4681]	4181	[4054; 4308]	− 368	− 8.1%	60.68	53.26	72.48	53.34

*CI* confidence interval, *CR* crude rate per 100,000 person-years, *ASIR* age-standardised incidence rate (European Standard Population) per 100,000 person-years.

^a^In the number of incident cases between 2025 and 2012.

^b^In incident cases between 2012 and 2025.

The age group (< 60 years) includes 0 to 60 years.

**Table 2:** Differences in the number of incident lung cancer cases in Catalonia between 2012 and 2025 by histology.

Lung cancer histology	Men						Women					
	2012		2025		Differences^a^	Net changes^b^	2012		2025		Differences^a^	Net changes^b^
	N	[95% CI]	N	[95% CI]	N	%	N	[95% CI]	N	[95% CI]	N	%
Non-small cell carcinoma	2556	[2457; 2655]	2287	[2193; 2381]	− 270	− 10.6%	600	[552; 648]	973	[912; 1034]	373	62.2%
Squamous cell carcinoma	1051	[987; 1115]	617	[568; 666]	− 434	− 41.3%	69	[53; 85]	64	[48; 80]	− 5	− 6.6%
Adenocarcinoma	1505	[1429; 1581]	1670	[1590; 1750]	165	11.0%	531	[486; 576]	909	[850; 968]	378	71.1%
Small cell carcinoma	420	[380; 460]	213	[184; 242]	− 207	− 49.3%	93	[74; 112]	87	[69; 105]	− 6	− 6.6%
Unspecified/ carcinoma NOS	649	[599; 699]	408	[368; 448]	− 241	− 37.2%	154	[130; 178]	162	[137; 187]	8	5.2%
Others	41	[28; 54]	20	[11; 29]	− 20	− 50.2%	36	[24; 48]	31	[20; 42]	− 5	− 13.4%
Total lung cancer	3666	[3547; 3785]	2928	[2822; 3034]	− 738	− 20.1%	883	[825; 941]	1253	[1184; 1322]	370	42.0%

*CI* confidence interval, *NOS* not otherwise specified. *Others* contains several histological types of low frequency.

^a^In the number of incident cases between 2025 and 2012.

^b^In incident cases between 2012 and 2025.

**Figure 3:**
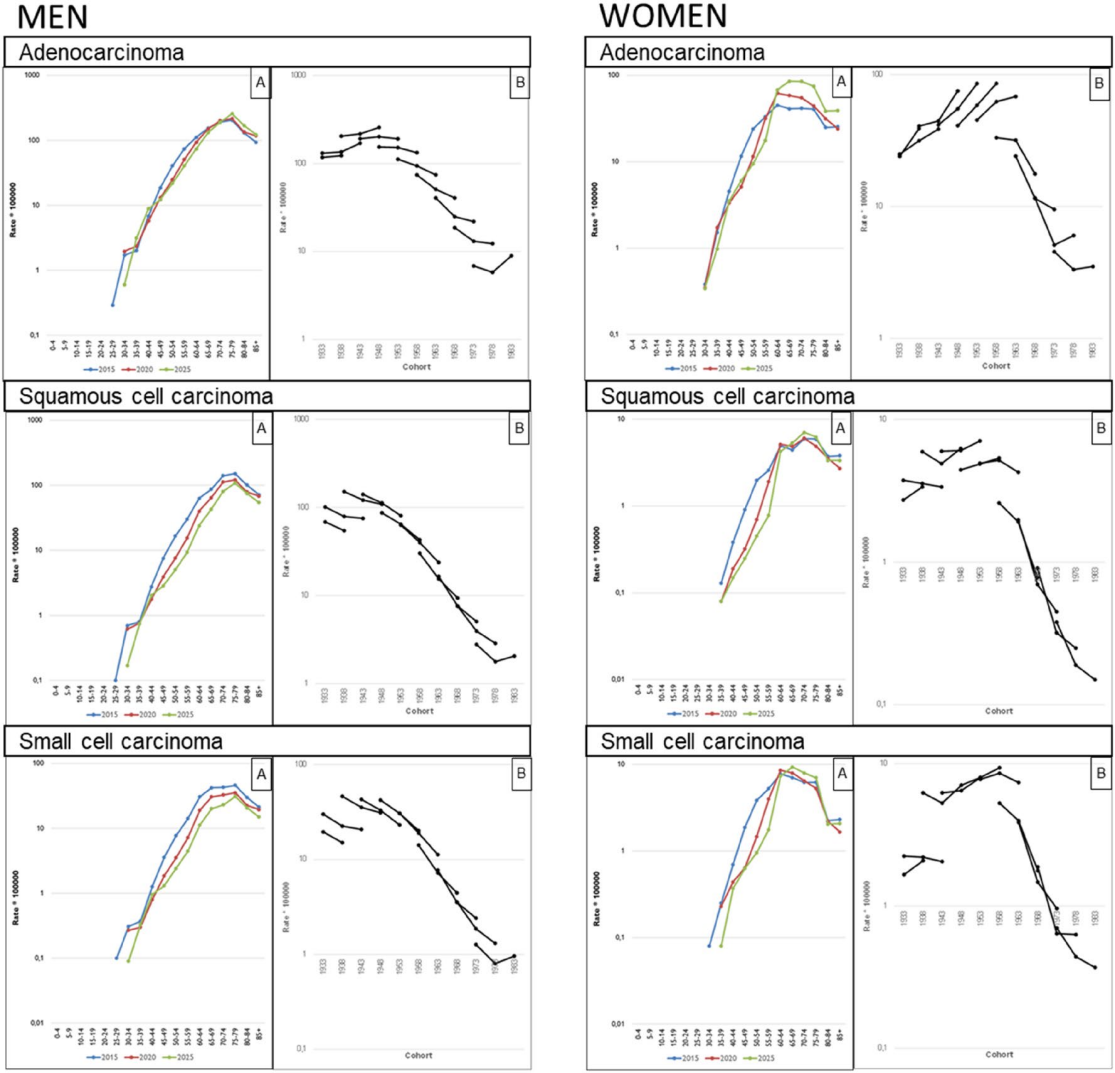
Time trends and projections of lung cancer by histology type in Catalonia up to 2025: (**A**) Age-specific incidence rates of lung cancer in 2020 (red) and 2025 (green) with respect to the reference year 2015 (blue). (**B**) Age-specific incidence rates by birth cohort. Note: These figures resulted from an age-period-cohort model by histological type and sex.

**Figure 4:**
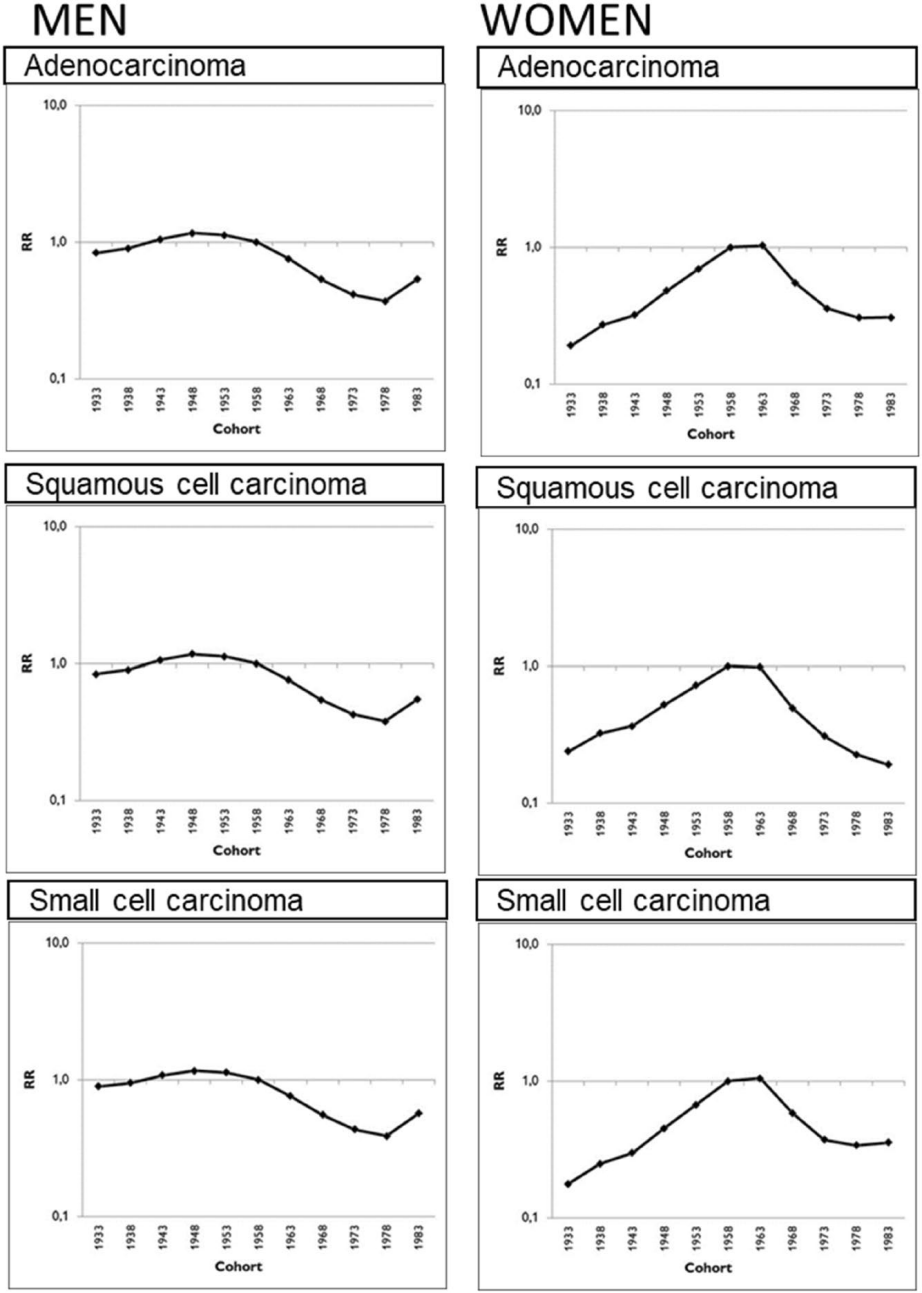
Relative risk of incidence by birth cohort for lung cancer by sex and histology in Catalonia for the 1931 to 1985 birth cohorts. Note: These figures resulted from a cohort model of effect of the birth cohort. Reference cohort: 1958 (1956–1960).

In order to establish the contribution of age, period and cohort in the observed incidence changes, we carried out an APC model using the estimated data of lung cancer incidence for Catalonia in the years 2015, 2020 and 2025, using the quinquennial cohorts of those born from the 1931–1935 period to the 1981–1985 period, and the five-year age groups from 35–39 years to 80–84 years and 85 or more years. The Poisson APC model [ln (Cases/Population) ~ Factor(Age) + Continuous(Drift) + Factor(Period) + Factor(Cohort)], was estimated by NORDPRED R function^16^ which is the most commonly software package used among the studies using APC models^17^. Due to collinearity among age, period and cohort, linear effects of period and cohort cannot be estimated simultaneously, a common linear trend ('drift') is estimated instead. The graphs resulting from a cohort model of age period by histological type and sex (Fig. 3) and the effect of the birth cohort (Fig. 4) are also shown. The CR and relative risk of the components of the APC model between 2015–2025 are presented in the Supplementary material Table 1.

### Ethics declaration

Authors confirm that all methods were carried out in accordance with relevant guidelines and regulations.

## Results

Table 1 presents the projected incidence of lung cancer in Catalonia between 2012 and 2025 by sex and age group. There were an estimated 4549 new cases of lung cancer in 2012 (N = 3666 in men and N = 883 in women). From 2012 to 2025, incident cases are expected to decrease by 20.1%, to 2928 new cases in men, mainly due to a decrease in the youngest age groups (< 60 years, − 53.7%; 60–69 years, − 34.2%). The pattern for women is different, showing a projected overall increase of 42.0% (from 883 to 1253), concentrated in the groups aged 60 to 69 years (+ 136.9%) and 70 to 79 years (+ 115.7%), in contrast with an expected decrease of 57.0% in women under 60. In both sexes, the incident cases are observed to increase by 16% (from 2119 to 2458) for patients over 70 years.

Table 2 and Fig. 1 show the projected number of incident cases and trends in lung cancer by histological type. Adenocarcinoma, which was the most common type in both sexes, shows an expected increase of 11.0% in men and 71.1% in women. The overall increase in incidence of this histological type is largely accounted for by the rising incidence in women aged 60 to 79 years (Fig. 2). Squamous cell carcinoma and small cell carcinoma present respective decreases of 41.4% and 49.3% in men, and of 6.6% for both histological types in women.

Figure 3A shows the projected incidence rates by age, sex, and histological type in 2015, 2020, and 2025. Adenocarcinomas show a decrease from 2015 to 2020 and 2025 in men aged up to 60 years and in women in younger age groups. For squamous cell carcinoma and small cell carcinoma, the incidence rate is expected to decrease in men of all age groups in 2020 and 2025, whereas the data project an increase by 2025 in women aged 60 or older. If we consider the analysis by year of birth (Fig. 3B), the incidence rate shows an increase in adenocarcinomas in men born before the 1950s, while in younger cohorts, the rates decrease gradually and eventually stabilize. A similar trend is apparent in women, although the incidence rate only starts to decrease in the cohorts born after 1960. In squamous cell carcinoma and small cell carcinoma, the incidence rates consistently decrease with the age of the cohort; for women, this pattern is seen only in those born after 1960.

Men born before 1960 showed a higher relative risk of incident lung cancer for all histological types. In contrast, women at the highest relative risk were those born from 1958 to 1963 (Fig. 4).

## Discussion

Lung cancer trends show a strong divergence by sex and age group as well as marked differences in histology. In men under the age of 70, incidence is clearly decreasing in absolute terms, and projections show that this trend will be sustained until at least 2025. In women, this decline is only seen in those under 60. Decreased incidence is most evident for the histological types that are closely associated with smoking: in squamous cell carcinoma and small cell carcinoma in men. Adenocarcinoma is the dominant histological type in both sexes, but in women it accounts for virtually all of the rise in incidence.

Smoking is considered the most relevant risk factor for lung cancer, although with differences in terms of the relative risk for current smokers (> 30 cigarettes per day): for adenocarcinoma, odds ratio (OR) 10.8, 95% confidence interval (95% CI) 8.7–13.3 in men and OR 4.2, 95% CI: 3.5–5.0 in women; for squamous cell carcinoma, OR 45.6, 95% CI: 34.3–60.6 in men and OR 13.6, 95% CI 10.5–17.7 in women; and for small cell carcinoma, OR 45.7, 95% CI: 29.9–70.0 in men and OR 21.7, 95% CI: 15.5–30.1 in women^18^. Early initiation of smoking is associated with heavy smoking later on, lower cessation rates, and higher nicotine dependence in adulthood^19^. The number of cigarettes smoked per day (OR: 53.7, 95% CI 44.1–65.3 in men and OR: 40.2, 95% CI 23.7–68.1 in women > 30 cigarettes/day versus to never smokers) and the duration of smoking (OR: 32.8 95% CI 27.2–39.7 in men and OR: 12.2, 95% CI 8.7–17.1 in women who had smoked 50–59 years versus never smokers) are strongly associated with a much higher risk of smoking-related cancer^12,18,20^. From this perspective, it seems that the reduction in smoking observed in men in Catalonia and Spain in the late 20^th^ and early twenty-first century has had the expected impact on overall cancer incidence^21–23^. Smoking patterns are presented in the Supplementary Material Figs. 1, 2 and 3. In men over 70, the stabilization in the number of new cases is attributed to the peak in smoking prevalence rates in 1980 (68.0%; 95% CI: 66.1–69.9% in the 1950–1959 birth cohort)^21^, which was followed by a slow decrease until 2020 (< 30% for each age group)^23^. In contrast, the increased burden in women over 60 is primarily due to changes in smoking patterns dating back to 1970, when prevalence rates in women started to rise, with the highest prevalence found in 1990 (49.9%; 95% CI: 48.1–51.7% in the 1960–1969 birth cohort)^21^. Smoking prevalence rates in women slowly decreased between the 2000s to 2020 (approximately 20% for each age group), approaching those in men in all cohorts^23^. Consistent with the pattern of smoking^22^, our study found a higher risk of incident lung cancer in women born in the 1960s.

Moreover, our results suggest that the proportion of elderly patients newly diagnosed with lung cancer may increase by 16% in both sexes in the coming years, becoming a relevant part of daily oncological practice in Catalonia. Diagnosis and treatment in this group of patients are associated with specific challenges due to physiological changes in aging organs and usually, the presence of comorbidities^12,24,25^. These complexities require an individualized approach to therapeutic decision-making, potentially including a comprehensive geriatric assessment^26^.

Trends in lung cancer are generally associated with an increased incidence of adenocarcinoma. While this is true for both sexes, the growth is concentrated in women over the age of 60, in whom incidence rates are on the rise. Adenocarcinoma is the most prevalent histological type diagnosed in non-smokers, with potentially higher proportions occurring in women^18^. However, all histological types are associated with smoking, with the association strongest for squamous and small cell carcinoma and more modest for adenocarcinoma^18,20^. In our study, the increase in this histological type probably reflects the increased risk of adenocarcinoma in smokers due to changes in the design and composition of cigarettes^6,7,27^. While the evidence is not sufficient to specify which design changes are responsible for the increased risk of adenocarcinoma, it does suggest that ventilated filters, lower tar and nicotine-containing cigarettes could promote deeper inhalation, leading to more peripheral distribution of tobacco smoke in the lung^6^. Increased levels of tobacco-specific nitrosamines have also played a role^6,7^. The more rapid increase in relative risk associated with increasing smoking duration for squamous cell carcinoma and small cell carcinoma compared to adenocarcinoma, and the faster decrease after quitting^6,18^, could explain the similarity of the relative risk incidence trends of our study. Improvements in histological classifications and diagnostic techniques, especially of adenocarcinoma in concert with the identification on specific oncogenic factors (*EGFR* mutations or rearrangements of the *ALK*) have been suggested to affect their increase^20,27^. In addition, other factors that could modestly contribute to adenocarcinoma rates in both sexes could be related to air pollution, specifically nitrogen oxides^6,27^. However, these factors probably do not explain the magnitude of the observed changes.

The response to a given drug changes according to each histology, so it is crucial to identify the tumor characteristics in order to apply the most appropriate therapeutic approach^12^. Adenocarcinomas in non-smokers have some distinctive features arising from gene alterations, including a lower mutational load compared to other lung cancer histologies and oncogenic factors such as *EGFR* mutations or rearrangements of the *ALK* or *ROS1* genes^28–30^. These alterations are predictive biomarkers for the pathology and excellent therapeutic targets^12,29^. In contrast, immunotherapy has a lower clinical benefit for this type of tumor and is usually indicated in subsequent lines of treatment^12,29^.

The estimated incidence of squamous cell carcinoma and small cell carcinoma shows a downward trend from 2012 to 2025, which is more pronounced in men. Likewise, the overall incidence rate is projected to decrease by 2025 relative to 2015 and 2020. Tumors with a high mutational load usually have an etiological association with exposure to certain mutagens, as is the case of tobacco carcinogens in lung cancer^28,31^. In smokers, squamous cell carcinoma has a high number of somatic mutations, associated with increased immunotherapeutic activity that favors the clinical benefit of these treatments in such patients^28–30^. The recent introduction of anti-PD-L1 / PD-1 drugs has resulted in an increase in the overall survival rates of these patients^12,29^. Small cell carcinoma is one of the immunogenic tumors with the highest rate of somatic mutations due to exposure to tobacco^30^. However, the pathogenesis of this type of tumor presents an immunosuppressive phenotype affecting innate immunity and T cells, which hinders the activity of immunotherapy and could explain the negligible clinical benefits observed to date^9,32,33^. The function of different biomarkers, such as the PD-L1 expression percentage and mutational load, is being studied for the selection of patients who would benefit the most from immunotherapy^9,32^.

Our results on incidence trends in lung cancer are similar to those observed elsewhere in Spain. Izarzugaza et al.^28^ and other European countries such as Austria, France, Iceland, Italy, the Netherlands, and Switzerland^6^, argued that lung cancer incidence rates reflect the progress in smoking cessation, first observed in men and then also in women. The Cancer Incidence in Five Continents series reports data related to lung cancer histologies from 1998 to 2002^31^, showing that the proportion of adenocarcinomas is greater in women than in men in Spain, Italy, France, Germany, Switzerland, Portugal, Denmark, Belgium, and Austria. In most European countries, it also reflects the dominant pattern of decreasing squamous cell carcinoma in men in recent years^31^. These incidence trends concerning histological types are consistent with our findings.

Our study has some limitations. Cancer incidence data for the whole region are not available, so we assumed a homogeneous rate throughout Catalonia based on aggregated cancer incidence rates from Girona and Tarragona, provinces that together account for 20% of the Catalan population. In addition, the method we used is based on projecting the most recent trends into the future through future population estimates. However, changes in the most recent trends as well as in future population counts and age distributions could significantly alter the accuracy of these predictions. In the APC model, the number of squamous cell carcinomas and small cell carcinomas are very low and could compromise the power of the statistical analysis. It is also difficult to establish a relationship between the evolution in lung cancer incidence and changes in smoking patterns and types of tobacco consumed. One future area of study could focus on the impact of smoking electronic cigarettes on the development of malignant tumors.

## Conclusion

This study demonstrates that the changes in lung cancer incidence rates will have an important impact on the number of new cases by sex in elderly patients in the coming years, while the rising prominence of adenocarcinomas also has treatment implications. Both findings should be taken in account in resource planning for multidisciplinary teams that should include geriatric specialists.

## Supplementary Information

Supplementary Information.
