# Supplementary material for: Trends in lung cancer incidence by age, sex and histology from 2012 to 2025 in Catalonia (Spain)
Source: Sci Rep. 2021 Dec 2;11:23274. doi: 10.1038/s41598-021-02582-8 (PMC8639747; doi:10.1038/s41598-021-02582-8)
Supplement: Supplementary file 1 — Supplementary Information. [file 41598_2021_2582_MOESM1_ESM.docx]

**Supplementary material**

**TITLE:** Trends in lung cancer incidence by age, sex and histology from 2012 to 2025 in Catalonia (Spain)

**Authors:** Laura Guarga^1,2^, Alberto Ameijide^3^, Rafael Marcos-Gragera^4,5,6,7^, Marià Carulla^3^, Joaquim Delgadillo^8^, Josep Maria Borràs^4,9^, Jaume Galceran*^3,10^

^1^ Servei Català de la Salut (CatSalut), Barcelona, Spain.

^2^ Departament de Farmacologia, Universitat Autònoma de Barcelona, Cerdanyola del Vallès, Barcelona, Spain.

^3^ Registre de Càncer de Tarragona, Servei d’Epidemiologia i Prevenció del Càncer, Hospital Universitari Sant Joan de Reus, IISPV, Reus, Tarragona, Spain

^4^ Pla Director d’Oncologia, Departament de Salut, Barcelona. Spain

^5^Unitat d’Epidemiologia i Registre del Càncer de Girona (UERGG), Institut d’Investigació Biomèdica Girona Josep Trueta (IDIBGI), Girona (Spain)

^6^Biomedical Network Research Centers of Epidemiology and Public Health (CIBERESP), Madrid, Spain

^7^ Departament d’Infermeria, Universitat de Girona (UdG), Girona, Spain

^8^ Banc de Sang i Teixits (BST), Barcelona Spain.

^9^ Departament de Ciències Clíniques, Universitat de Barcelona, Campus de Bellvitge, L’Hospitalet de Llobregat, Barcelona, Spain

^10^ Departament de Medicina i Cirurgia, Universitat Rovira i Virgili, Reus, Tarragona, Spain

**Corresponding author:** Jaume Galceran. [jgalceran@epicancer.cat](mailto:jgalceran@epicancer.cat)

**Supplementary material table 1**. Crude rates and Relative risk of the components of the APC model of Lung incidence cancer in Catalonia. 2015-2025

|  |  | **MEN** |  |  | **WOMEN** |  |
| --- | --- | --- | --- | --- | --- | --- |
|  | **Squamous cell carcinoma** | **Adenocarcinoma** | **Small cell carcinoma** | **Squamous cell carcinoma** | **Adenocarcinoma** | **Small cell carcinoma** |
| Age | CR | CR | CR | CR | CR | CR |
| 35-39 | 1.8 (1; 2.8) | 3.2 (2.5; 4.1) | 0.8 (0.3; 1.6) | 0.2 (0; 0.5) | 1 (0.6; 1.5) | 0.2 (0; 0.6) |
| 40-44 | 5.8 (3.2; 10) | 10.8 (8; 14.4) | 2.8 (1.1; 6.3) | 0.4 (0; 1.7) | 2.2 (1.3; 3.4) | 0.4 (0.1; 1.4) |
| 45-49 | 11.9 (7.1; 19.2) | 22 (16.9; 28.6) | 6 (2.7; 12.3) | 0.5 (0.1; 2) | 3.5 (2.2; 5.1) | 0.7 (0.1; 2.1) |
| 50-54 | 18.3 (11.6; 27.9) | 34.2 (26.9; 43.2) | 9.4 (4.6; 17.7) | 0.6 (0.1; 2) | 4.3 (3; 6.1) | 0.8 (0.2; 2.3) |
| 55-59 | 25.8 (17.5; 36.9) | 48.8 (39.6; 59.8) | 13.4 (7.3; 23.2) | 0.8 (0.2; 2.2) | 6 (4.3; 8.1) | 1.2 (0.3; 2.9) |
| 60-64 | 48.1 (34.9; 64.9) | 65.9 (55; 78.7) | 25.8 (15.5; 41.1) | 2.1 (0.7; 5) | 12 (9.1; 15.7) | 2.6 (0.9; 5.8) |
| 65-69 | 65.9 (50.8; 84.3) | 90.4 (77.3; 105.6) | 35.4 (23.2; 52.5) | 2.6 (1.1; 5.5) | 15.8 (12.4; 19.9) | 3.5 (1.4; 7.3) |
| 70-74 | 113.7 (92.4; 138.9) | 118.1 (102.5; 135.7) | 37.5 (26.4; 52.7) | 4.7 (2.4; 8.9) | 22.5 (18.2; 27.6) | 4.5 (2.1; 8.7) |
| 75-79 | 141.7 (119.5; 167.7) | 147.4 (129.2; 167.9) | 46 (33.8; 62.2) | 5.6 (3.2; 9.5) | 27.7 (22.9; 33.4) | 5.6 (2.9; 10.6) |
| 80-84 | 106.2 (90.1; 124.9) | 106.1 (92.6; 121.1) | 32.6 (24; 44) | 4.5 (2.6; 7.6) | 22.6 (18.7; 27.3) | 2.7 (1.3; 5.2) |
| 85+ | 95.8 (82.1; 111.4) | 94.9 (83.7; 107.1) | 28.9 (21.9; 37.5) | 4.8 (3; 7.5) | 25.5 (21.5; 30) | 3 (1.7; 5.1) |
| Period | RR | RR | RR | RR | RR | RR |
| 2015 | Ref | Ref | Ref | Ref | Ref | Ref |
| 2020 | 0.72 (0.64; 0.8) | 0.95 (0.88; 1.01) | 0.7 (0.58; 0.83) | 0.73 (0.49; 1.03) | 0.92 (0.83; 1.02) | 0.72 (0.49; 0.95) |
| 2025 | 0.56 (0.48; 0.64) | 0.98 (0.9; 1.05) | 0.54 (0.43; 0.67) | 0.63 (0.37; 0.93) | 0.99 (0.88; 1.11) | 0.6 (0.36; 0.84) |
| Cohort | RR | RR | RR | RR | RR | RR |
| 1933 | 0.83 (0.71; 0.98) | 0.84 (0.74; 0.96) | 0.89 (0.67; 1.2) | 0.24 (0.14; 0.41) | 0.19 (0.16; 0.23) | 0.18 (0.09; 0.34) |
| 1938 | 0.9 (0.75; 1.1) | 0.9 (0.79; 1.03) | 0.95 (0.69; 1.33) | 0.32 (0.18; 0.62) | 0.27 (0.23; 0.33) | 0.25 (0.13; 0.5) |
| 1943 | 1.05 (0.83; 1.36) | 1.06 (0.91; 1.24) | 1.08 (0.73; 1.64) | 0.37 (0.18; 0.88) | 0.32 (0.26; 0.41) | 0.3 (0.14; 0.72) |
| 1948 | 1.17 (0.87; 1.6) | 1.17 (0.98; 1.4) | 1.17 (0.74; 1.93) | 0.52 (0.22; 1.58) | 0.48 (0.37; 0.64) | 0.45 (0.2; 1.33) |
| 1953 | 1.13 (0.79; 1.65) | 1.13 (0.92; 1.39) | 1.13 (0.66; 2.07) | 0.73 (0.26; 2.79) | 0.7 (0.52; 0.97) | 0.67 (0.27; 2.47) |
| 1958 | Ref | Ref | Ref | Ref | Ref | Ref |
| 1963 | 0.76 (0.47; 1.27) | 0.76 (0.58; 0.99) | 0.76 (0.37; 1.71) | 0.99 (0.25; 6.23) | 1.03 (0.7; 1.58) | 1.05 (0.33; 6.1) |
| 1968 | 0.54 (0.31; 0.97) | 0.55 (0.41; 0.74) | 0.55 (0.25; 1.39) | 0.5 (0.11; 4.04) | 0.55 (0.35; 0.89) | 0.58 (0.16; 4.31) |
| 1973 | 0.41 (0.23; 0.8) | 0.43 (0.31; 0.59) | 0.43 (0.18; 1.21) | 0.31 (0.06; 3.22) | 0.36 (0.22; 0.62) | 0.37 (0.09; 3.49) |
| 1978 | 0.37 (0.2; 0.75) | 0.38 (0.27; 0.54) | 0.39 (0.15; 1.17) | 0.23 (0.04; 2.86) | 0.31 (0.18; 0.55) | 0.34 (0.08; 3.97) |
| 1983 | 0.54 (0.26; 1.18) | 0.55 (0.38; 0.81) | 0.57 (0.19; 1.94) | 0.19 (0.02; 3.32) | 0.31 (0.17; 0.59) | 0.36 (0.06; 5.41) |

CR: crude rate; RR; relative risk

**
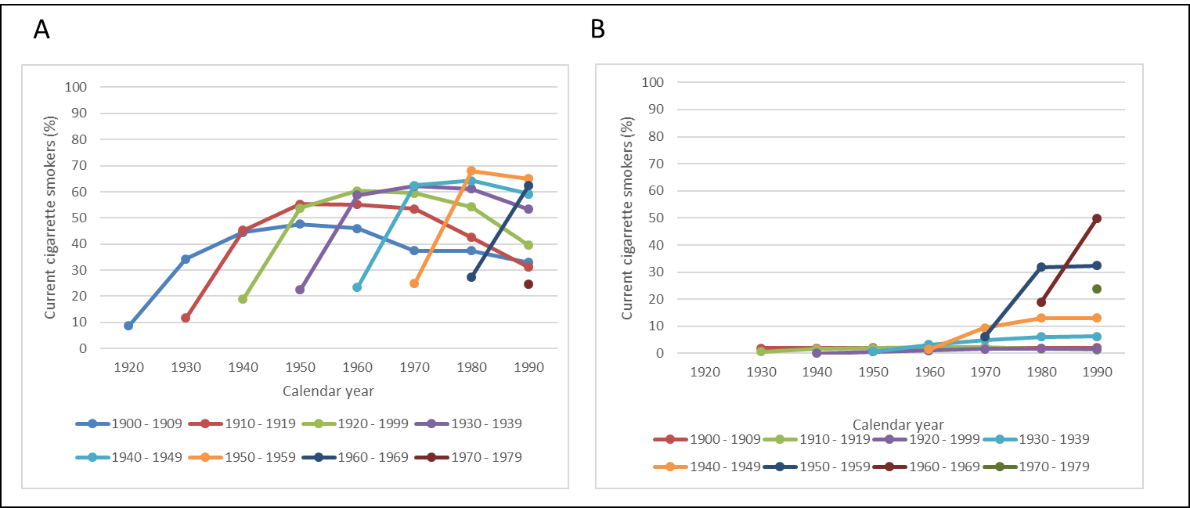
**

**Supplementary material Figure 1**. Estimated prevalence of current cigarette smoking among successive birth cohorts of men (A) and women (B), Spain, 1910-1990.

*Note: The figure was adapted from Fernandez, et al. European Journal of Cancer Prevention. 2003 (reference #21). Reflect trends in the in the percentage (%) of smokers (current cigarette smokers, at least one cigarette per day and occasional smokers) from the Spanish National Health Interview Surveys (NHIS), a cross-sectional survey conducted for a representative sample Spanish population*

**
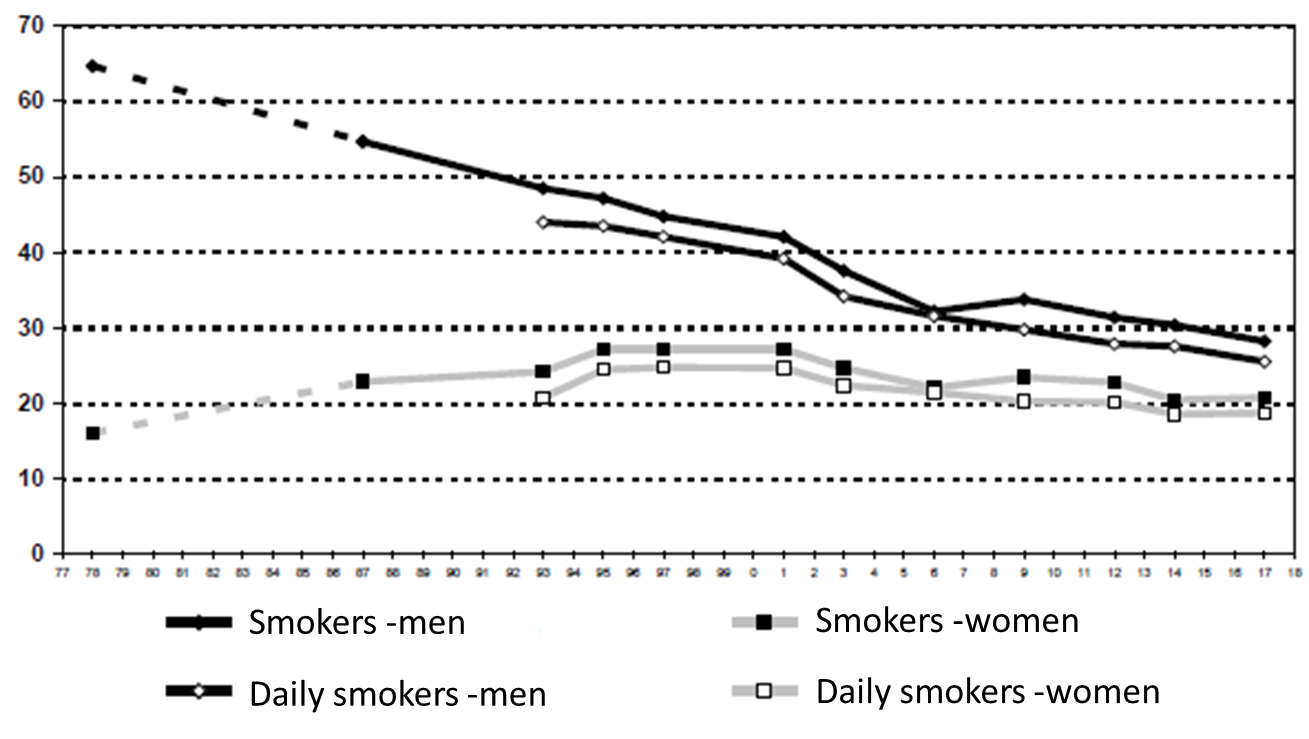
**

**Supplementary material Figure 2**. Estimated prevalence of smokers by sex, Spain, 1978-2017.

*Note: The figure was adapted fromVillalbí, Rev Esp Salud Pública. 2019 (reference #22). Reflect trends in the percentage (%) of smokers (include daily smokers, at least one cigarette per day and occasional smokers) from the Spanish NHIS, a cross-sectional survey conducted for a representative sample Spanish population*

**
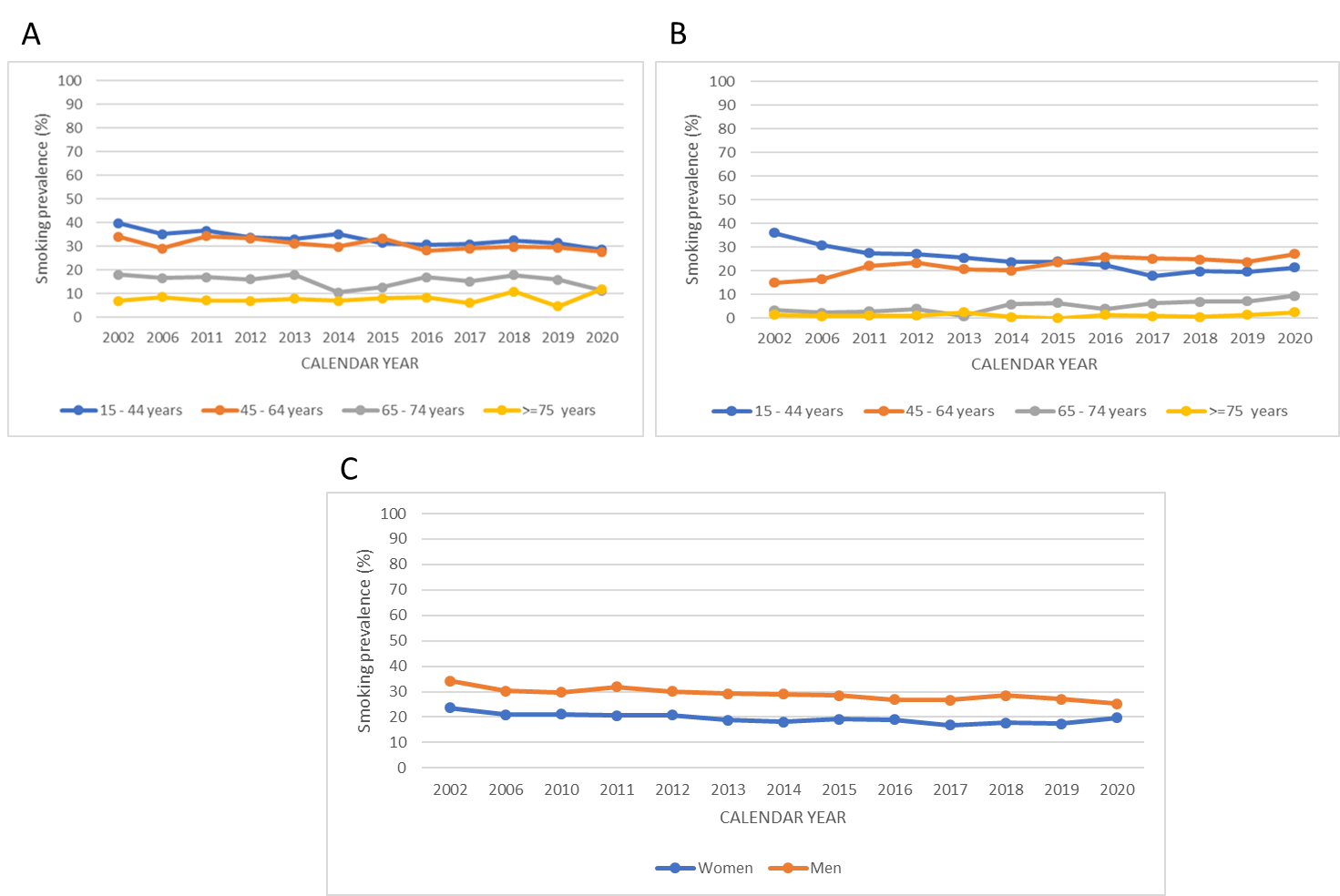
**

**Supplementary material Figure 3**. Estimated prevalence of current cigarette smoking by age groups of men (A), women (B) and all age groups (C), Catalonia, 2002-2020.

*Note: Data were obtained from the Catalan Health Interview Survey, a cross-sectional survey conducted for a representative sample Catalan population:* <https://salutweb.gencat.cat/ca/el_departament/estadistiques_sanitaries/enquestes/esca/resultats_enquesta_salut_catalunya/> *(reference #23). These figures reflect trends in the percentage (%) of current smokers (include daily smokers, at least one cigarette per day). Data from 1994 was not considered in figure A, B or C because they was published considering different smoking categories. Data from 2010 was not considered in figure A and B because they were not published by age groups.*
